# A Small-Pox Preventive

**Published:** 1882-03

**Authors:** 


### A SMALL POX PREVENTIVE.

JANE G. SWISSHELM, a lady who has had a great deal of experience in hospitals, has a new remedy for the small-pox, viz., the daily use of fruit acids. She says :

"From the magical effects of lemons, dried apple sauce, and citric acid in hospital gangrene; from the almost certainty with which hard cider cures scarlet fever and diphtheria, it occurs to me that small-pox, being also a kind of blood poison, might be met with fruit acids. One physician has published an account of treating himself successfully with lemons—another recommends cream of tartar tea. The principle in every case is the acid, and in connection with simple, wholesome nourishment and pure air it appears to me invincible in all that class of diseases in which there is decomposition of blood. I doubt if people who sleep in well ventilated rooms and eat fruit at every meal are liable to small-pox, scarlet fever, or diphtheria; and when one does take either, fruit acid must be an important item in the treatment of the case."

Mrs. Swisshelm thinks it would be well for boards of health to gather statistics, and see how many fruit-eaters take small-pox as compared with the consumers of pork, and recommends that every table should be supplied with baked apple, apple-sauce, or some other kind of acid fruit in simple form—fruit that has not been preserved in tin cans. With this precaution, the liability to disease would, in her opinion, be largely diminished. This is certainly an easy remedy and an exceedingly pleasant one, and is worthy a trial.

### CAPTURED BY BRIGANDS.

Mr. Suter has given to a correspondent of the London *Standard* at Salonica an account of the experiences he had while a captive of the brigands :

"On the night of the capture, immediately after separating from his wife, he was hurried off to a distance of twelve miles from Isvor, where he was hidden all the following day. For nearly a fortnight his hiding-place was changed every night. His arms were kept bound, and two men were always standing over him with loaded rifles for two days after he was taken. The brigands were composed of two bands—one of thirteen, under Captain Aristidi and Ghiorghi Katzaro; the other of twelve, under Captain Nicola. Some of the brigands were Ottoman Greeks, some Hellenes, others Christian Albanians and deserters from the Greek army. There was always great discord between the two bands, and in their quarrels the captive's life was often in great danger. Mr. Suter and his captors slept on leaves upon the ground. The days were mostly spent in dancing, drinking and singing when the brigands were not absent on foraging expeditions, and part of their time was passed in playing cards and in telling stories of murders and exploits. The brigands used most profane and blasphemous language; but they were superstitious, and were strict in their religious observances. They fasted the whole of Lent, and celebrated Easter with great solemnity."

Then follows an account of the negotiations for and payment of the ransom leading to the release of Mr. Suter. The correspondent adds :

"During Mr. Suter's captivity sentries were always posted at advantageous positions. They wore capes to cover their arms. The brigands were all capital shots, and well armed. Some of their rifles were Martini's, of the Turkish pattern, and some were Chassepots with Messageries Imperiales marks, and one was of Greek pattern. The brigands had no difficulty in procuring ammunition. They were most suspicious of being poisoned. Whenever provisions were brought the peasants were forced to taste everything. They were well informed as to all that is passing in Salonica, and in the whole of Macedonia. They were supplied with Greek newspapers, and took a lively interest in the negotiations about the ratification of the Greek frontier."

---

A Sticking-Plaster.—An excellent sticking-plaster for fresh cuts or cracked hands is made of three pounds of resin, a quarter of a pound of beeswax, a quarter of a pound of mutton tallow. When well melted and dissolved together, remove from the fire and keep stirring till it is about as cool as it will pour. Then add about one tablespoonful of spirits of turpentine ; then pour the whole into a pail of cold water, and when cool enough take it out and work it as a shoemaker does his wax. When sufficiently worked roll it out in small sticks. This is equal to any plaster ever bought. Keep the hands greased, to prevent it from sticking to them while working it.

